# Calcite-dissolving bacteria: promising approach as bio-fertilizer

**DOI:** 10.3389/fmicb.2025.1533127

**Published:** 2025-07-24

**Authors:** Christina Lemson Hambo, Mawazo Jamson Shitindi, Kalunde Pilly Sibuga, Yasinta Beda Nzogela

**Affiliations:** ^1^Department of Crop Science and Horticulture, College of Agriculture, Sokoine University of Agriculture (SUA), Morogoro, Tanzania; ^2^Department of Biological Science, Faculty of Science, Dar es Salaam University College of Education (DUCE), Dar es Salaam, Tanzania; ^3^Department of Soil and Geological Sciences, College of Agriculture, Sokoine University of Agriculture (SUA), Morogoro, Tanzania

**Keywords:** calcite, calcite dissolving bacteria, calcareous soil, biofertilizer, calcium uptake, crop productivity

## Abstract

Calcium (Ca) is an essential macronutrient and a secondary messenger for the proper growth and functioning of plants. It is essential for membrane stability, cell integrity, cell division, and elongation. In the soils, Ca exists in inorganic and organic forms. Inorganic fraction constitutes soil-Ca solution, which is readily available for plant uptake, exchangeable Ca, which replenishes the solution pool, and fixed Ca, which replenishes exchangeable Ca slowly upon weathering to release calcium ion (Ca^2+^). Similarly, organic forms of Ca are inactive and unavailable for plant uptake until decomposed, mineralized, and dissolved into Ca^2+^. Calcium deficiency in soil reduces plant growth, development, and yields, which can be rectified by applying Ca fertilizers and Ca-rich soil amendments. Unfortunately, many smallholder farmers have limited access to Ca fertilizers, and thus cannot purchase optimal amounts required for enhancing plant growth and crop yields. This calls for alternative technologies that enhance the dissolution of unavailable forms of Ca in the soil. Calcite-dissolving bacteria (CDB) are a functional group that can dissolve poorly soluble calcite minerals into Ca^2+^, thus increasing the % Ca^2+^ saturation on the soil exchange sites, making it available for plant uptake. CDB offers an economically viable and environmentally friendly option to overcome Ca deficiency in the soil. CDB has been a subject of research interest, especially in its ability to precipitate calcite for soil stabilization and strength enhancement. However, studies on using CDB to improve the Ca^2+^ supply power of the soils and their resultant effects on plant growth and crop productivity, especially under field conditions, are limited. For effective formulation of CDB-based biofertilizers, one should understand the chemistry of calcite, Ca availability in the soil, diversity of CDB, mechanisms of calcite dissolution by CDB, mechanisms by which CDB promote plant growth, and the potential of CDB as biofertilizers in crop production. This review is among the first to provide detailed information on these aspects of CDB. We employed a Preferred Reporting Items for Systematic Review and Meta-Analysis (PRISMA) method to explore and expand the understanding of the potential of CDB as biofertilizers in crop production.

## 1 Introduction

Calcium (Ca) is among the major growth-limiting macronutrients required for proper plant growth, development, and productivity. It is important for maintaining membrane stability, cell integrity, cell division and elongation, thus playing vital roles in plant growth and development, imparting mechanical strength and resistance to insect pest damage and disease infestation in plants. In its ionic form, Ca plays crucial roles in stimulus-response reactions of cells as a second messenger (Edel et al., 2017). Calcium comprises 0.2%−0.8% of the dry weight of plants and is present in nucleic acids, enzymes, coenzymes, nucleotides, and phospholipids (Holland et al., 2018). Among all plant parts, leaves contain the highest concentration of Ca, mainly in the form of Ca pectate found in the middle lamella of cells (Holland et al., 2018; Jing et al., 2024). According to Holland et al. (2018), Ca aids the whole plant's transport systems, including water, nutrients, and sugar. It promotes cell division and elongation, signal transduction and plant responses, flower and fruit formation, and root development (Kuronuma and Watanabe, 2021). Furthermore, Ca is essential for cell wall integrity and development, offering structural support and a defense mechanism to the plant (Shibzukhov et al., 2021). According to Huber et al. (2011) and Jing et al. (2024), adequate supply of Ca in the plant tissue regulates plant disease occurrence via following mechanisms (i) Calcium ion (Ca^2+^) as a second messenger plays a key role in recognition of pathogenic invaders at the plasma membrane; (ii) Imparting stability to bio-membranes and cell walls, hence Ca deficiency increases the efflux of low-molecular weight compounds from the cytoplasm to apo-plasm; (iii) Formation of Ca-polygalacturonates in the middle lamella for cell wall stability. Ca is used to produce pectin, which holds the cells together and strengthens the cell wall, resulting in increased tissue firmness and inhibiting the activity of pectolytic enzymes from dissolving the middle lamella. These reduce the susceptibility of plant infections caused by parasitic fungi and bacteria. Furthermore, Ca aids in a plant's stress tolerance by enhancing the plant's ability to recover from different stresses and reducing stress-induced damages caused by salinity, drought, and temperature fluctuation (Jing et al., 2024).

Calcium deficiency causes different physiological disorders in plants. It primarily appears as localized tissue necrosis, causing stunted growth, curling leaves, and finally death of terminal buds and root tips (Chen et al., 2018). Calcium is phloem-immobile in the plant; therefore, its deficiency affects new tissue, normally the meristems (Chen et al., 2018). Crop-specific symptoms of Ca deficiency include tip burns in cabbage and lettuce, bitter pit in apples, blossom-end rot in tomato, watermelon, and pepper, as well as blackheart in celery, which strongly reduce crop quality and yield (Kuronuma and Watanabe, 2021).

Microbial communities play dynamic roles in mobilizing sparingly available nutrients from the soil, promoting plant growth, development, and stress responses. They play critical roles in the Ca cycle to produce soluble Ca for the plant's uptake by dissolving a less soluble form of Ca (calcite) to release Ca^2+^ ions, making it available to plants (Peper et al., 2022). Generally, calcite can be dissolved by different processes, including weathering, erosion, and biological activity (Tamilselvi et al., 2016). One of the most interesting aspects of calcite dissolution is the role played by calcite-dissolving bacteria (CDB), which dissolve poorly soluble calcite to generate Ca^2+^, and the plant can absorb it (Tamilselvi et al., 2016). The capability of CDB to dissolve calcite has also attracted numerous commercial procedures to use CDB as an effective means to dispose of calcite deposits from pipes and other unwanted calcite formations (Rana et al., 2015). Furthermore, calcite dissolution by CDB has been proposed as a substitute technique to reclaim calcareous sodic soils (Rana et al., 2015; Tamilselvi et al., 2016). Different studies have explored the ability of CDB to precipitate calcite for soil stabilization and soil strength enhancement (Mujah et al., 2017; Hadi and Saeed, 2022; Fu et al., 2023). However, limited studies are reporting on their use in calcite dissolution. Hereafter, this review aims to provide a detailed description of calcite, the availability of Ca in soils, the diversity of CDB, the mechanisms of Calcite dissolution by CDB, how CDB promotes plant growth, and the potential of CDB as biofertilizers in crop production.

## 2 Methodology for literature search

**Figure 1:**
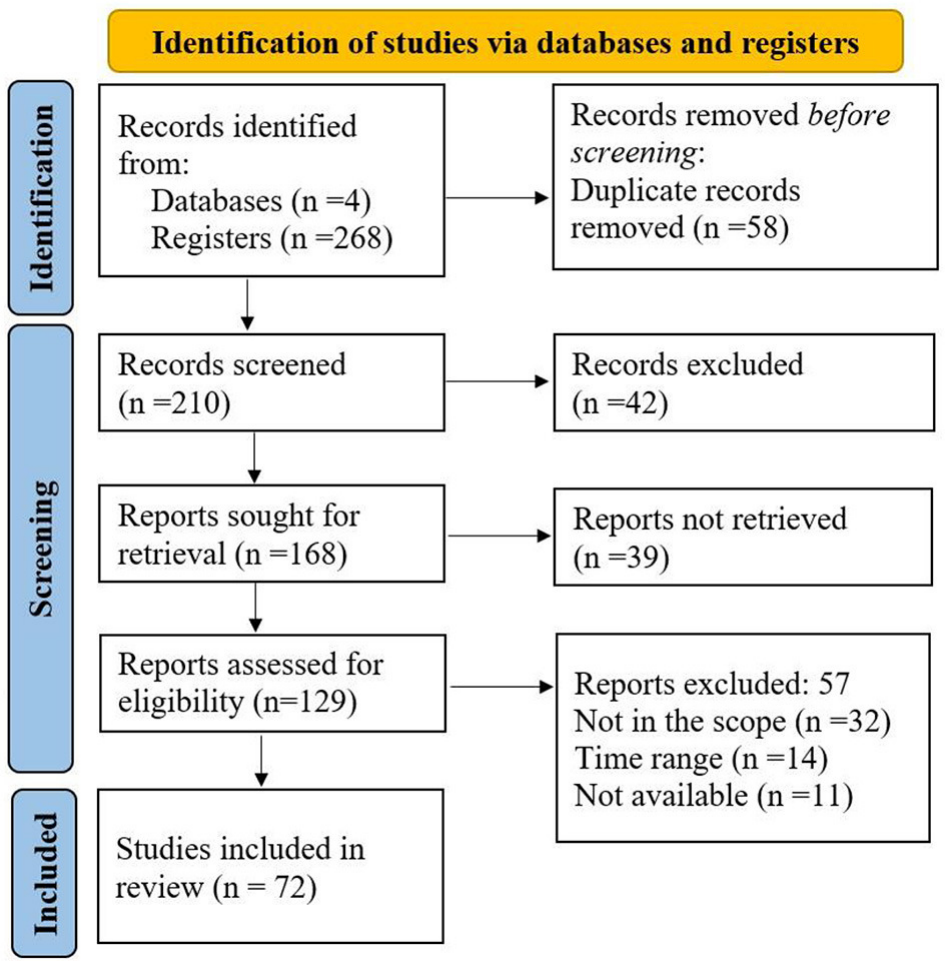
The PRISMA flow chart employed in this review shows the identification of literature from the database, the screening process, and the literature included.

This review used the Preferred Review Items for Systematic reviews and Meta-Analyses (PRISMA) method (Page et al., 2021) to explore the diversity of calcite-dissolving bacteria (CDB), mechanisms of calcite dissolution, the promotion of plant growth by CDB, and trends and prospects of using CDB as biofertilizers. The PRISMA technique ensures that the literature is sufficiently covered and suggests checklists for addressing vital topics, as shown in Figure 1 (Page et al., 2021). To search for the relevant publications that fit the study's scope, data on calcite dissolution were collected from four sources, including Google Scholar, Research Gate, Web of Science, and Science Direct. The search terms used were “Calcite dissolving bacteria,” “Calcite dissolution,” “Calcite solubilization,” and “Soil calcium.” The terms were used to search in “Title”, “Abstract,” and “Keywords” of the articles to retrieve relevant research outputs, published reviews, and reports from the databases. Research articles authored in English were retrieved from the database from 2000 to 2024. The search yielded 268 papers: 136 from Google Scholar, 78 from Web of Science, 45 from Science Direct, and nine from Research Gate. Papers were revised, and duplicates were ignored for the authentic evaluation process. Results from these sources have been reported in the subchapters discussing calcareous soil and availability of Ca in the soil, the diversity of CDB, mechanisms of CDB in calcite dissolution, plant growth promotion by CDB, trends, and prospects of CDB uses as biofertilizer.

## 3 Results of the review

**Figure 2:**
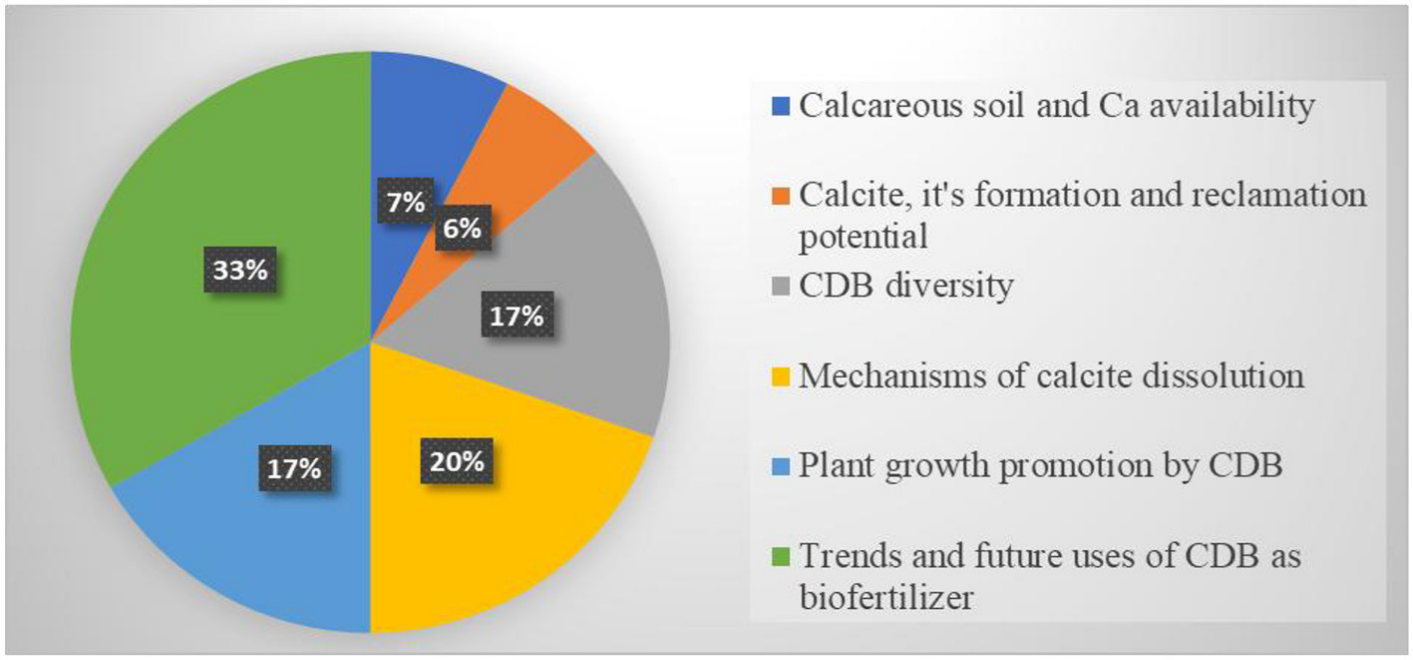
The Pie chart shows the distribution of literature used in the results.

The findings of this review have been presented and discussed below. The discussion focuses on calcareous soils and Ca availability, diversity of CDB, mechanisms of calcite dissolution, plant growth promotion by CDB, trends, and prospects of using CDB as biofertilizers. The distribution of studies involved in each section is presented in Figure 2. Of the 72 studies included in this review, 66 contributed to the results section. Descriptive statistics indicate that 7% of studies addressed calcareous soil and the availability of calcium in the soil, while 6% provided significant insights into calcite formation and its potential for reclamation. Furthermore, 17% of studies discussed the diversity of Calcite dissolving bacteria, 20% elucidated the mechanisms of calcite dissolution, 17% revealed plant growth-promoting effects of CDB, whilst 33% focused on the trends and prospects of CDB applications as biofertilizers. Based on this analysis, most studies focused on trends and prospects of using CDB as biofertilizers, highlighting the wide potential of microbial-based formulations as biofertilizers for enhancing fertility and crop productivity. This also indicates a progressive shift toward more biological approaches in soil and crop management practices, which could benefit the sustainability of agriculture.

### 3.1 Calcareous soil and availability of calcium in the soil

Calcareous soils are defined as soils containing a high amount of calcium carbonate (CaCO_3_), which distinctly affects both physical and chemical properties of the soil related to plant growth, including aggregate stability, water retention capacity, soil crusting, and availability of plant nutrients (Bolan et al., 2023). Calcareous soils are characterized by CaCO_3_ in the soil parent material and consequent accumulation of free CaCO_3_ in the soil profile (Taalab et al., 2019). Often, calcareous soils have more than 15% CaCO_3_ (6.15% Ca) in the soil, possibly occurring in various forms (White and Broadley, 2003). The primary source of soil Ca is from weathered limestone and other primary minerals. Different factors influence the concentration and distribution of Ca in the soil, including the nature of parent materials, climate, and pedogenesis (Holland et al., 2018; White and Broadley, 2003). Calcareous soils are generally alkaline, cover a significant global area, and are often found in arid regions (Holland et al., 2018). In contrast, non-calcareous soils contain limited amounts of free CaCO_3_, although such soils might contain large amounts of Ca, principally if Ca-fertilizers or liming materials have been applied (Holland et al., 2018).

The presence of Ca in soils can vary greatly and is closely related to the soil pH. Alkaline soils with soil pH above 7.0–8.5 usually have optimal Ca concentration for plant growth and development, whilst for acidic soils with pH below 6.0, the exchangeable Ca level in the soil is frequently low, and the solubility of Manganese (Mn) and Aluminum (Al) increases, which can be toxic (Holland et al., 2018). Most plant-available Ca exists in exchangeable form, usually much greater than Ca found directly in the soil solution. Holland et al. (2018) state that the average Ca concentration in soil solution is ~0.01 mol/L. For most soils, the approximate range of exchangeable Ca is 20%−65% of the cation exchange capacity (CEC) (Holland et al., 2018; Jing et al., 2024). Mineral Ca present in the soil can be in the form of precipitated calcite (CaCO_3_) or gypsum (CaSO_4_.2H_2_O) within the soil profile, which causes Ca concentrations to be much greater than the exchangeable (Han et al., 2019). However, Ca has fewer pathways than other macronutrients; thus, its management is easier. In the soil, Ca is primarily lost through plant uptake since it is not subject to volatilization, and leaching losses occur to a lesser extent (Holland et al., 2018; Jing et al., 2024; White and Broadley, 2003). Calcium is not easily leached because it is held to cation exchange sites much more strongly than other nutrient cations, as shown on the lyotropic series in Equation 1 (Han et al., 2019).


(1) $${\text{Al}}^{\text{3+}}>{\text{Ca}}^{\text{2+}}>{\text{Mg}}^{\text{2+}}>{\text{K}}^{\text{+}}{\text{=NH}}_{\text{4}}^{\text{+}}>{\text{Na}}^{\text{+}}$$

However, other factors lead to soil-Ca depletion by lowering the soil pH below 5.5. These factors include acid rain, mineralization of organic matter, weathering of parent materials, and the use of acidifying fertilizers, especially sulfur, Urea, and Ammonium-based fertilizers (Goulding, 2016). Hereafter, for proper soil Ca management, Ca inputs should be considered. Very little available Ca is derived from mineralization of soil organic matter; hence, input of available Ca is generally from weathering of Ca-containing minerals and supplemental applications of Ca-based fertilizers (Han et al., 2019). Addition of gypsum or lime is also important in raising soil-Ca level, as well as in adjusting soil pH (Goulding, 2016). Furthermore, the use of nitrate forms of nitrogen in soil is recommended rather than urea and ammonium forms, since excess ammonium reduces Ca uptake in soil by increasing Mn and Al solubility (Kuronuma and Watanabe, 2021).

### 3.2 Formation of calcite and its potential for reclamation

Calcite is a rock-forming mineral with a chemical formula of CaCO_3_. It's the most common and abundant mineral on Earth, making up ~4% of the Earth's crust (Gao et al., 2017). It's the primary component of limestone and marble, defining hardness 3 on the Mohs scale of mineral hardness (Morse et al., 2007). Calcite formation is a complex process influenced by different geological conditions, such as temperature, pressure, and fluid composition. Limestone, a calcite-sedimentary rock, is formed through the accumulation of mineral and organic material over a period. In the marine environment, limestone is formed through the accumulation of shells and skeletons of marine organisms like plankton, mollusks, and algae, a process known as biomineralization (Pastore et al., 2022; Wei et al., 2015).

Metamorphic rocks such as marble are formed through the recrystallization of limestone due to high temperature and pressure. During this process, the calcite crystals in the limestone and dolostone undergo changes in their crystal structure and orientation under high temperature and pressure, forming a distinctive texture and appearance of marble (Morse et al., 2007; Petrash et al., 2017). Calcite can also be formed by precipitation from groundwater in caves, forming stalactites and stalagmites (Wei et al., 2015).

Calcite has several potential applications in land reclamation, environmental remediation, and industrial activities. In agriculture, calcite is an important soil amendment, used as liming material to neutralize soil acidity and enhance soil pH balance, while acting as a source of Ca for plant growth (Gao et al., 2017; Young Nam, 2003). Calcite also plays an important role in Carbon Capture and Storage (CCS) technologies, where the process of limestone formation removes carbon dioxide from the atmosphere and stores it for a long time, hence reducing greenhouse gases (Morse et al., 2007). It's further used in the construction industry for cement and concrete production as well as in architecture and sculpture for its aesthetic qualities (Gao et al., 2017). Concretes made from calcite are used to construct bridges, highways, buildings, and other important structures. Although not documented by studies in this review, calcite can serve as a raw material or ingredient in the formulation of Ca-containing fertilizers, taking advantage of its high Ca content.

### 3.3 Diversity of calcite-dissolving bacteria

Calcite Dissolving Bacteria refers to a functional group of bacteria that can break down and dissolve calcite minerals, making Ca and other constituent nutrient elements available for plant uptake (Peper et al., 2022). These bacteria are found in a wide range of environments, including calcareous soils, freshwater bodies, animal wastes, farmlands, oceans, and limestone quarries (Subrahmanyam et al., 2012; Rana et al., 2015; Tamilselvi et al., 2016). They play a crucial role in the biogeochemical cycling of Ca and other essential environmental nutrients. Microorganisms in soil are the most plentiful; there are more soil microbes in one teaspoon of soil than there are people on Earth (Backer et al., 2018; Kalayu, 2019). An area of 1 m^2^, at a depth of 15 cm, might contain 500 g of bacteria, 500 g of actinomycetes, and 1.5 kg of fungi, depending on the type of ecosystem (Backer et al., 2018). Generally, 1 g of fertile soil contains 101–1,010 bacteria, and their live weight may exceed 2,000 kg ha^−1^, among which some have calcite dissolution activity (Kalayu, 2019). Among 12 studies reporting on CDB diversity in this review work, 29% reported on *Bacillus* sp., while *Pseudomonas* sp. and *Staphylococcus* sp. were reported by 13% of studies each. Other bacterial strains to have calcite dissolving effect, with the percentage of studies reporting their effect on calcite dissolution in brackets, were *Paenibacillus* sp. (10%), *Brevibacterium* sp. (10%), *Buttiauxella* sp., and *Azotobacter* sp. (6%), while *Enterobacter* sp., *Cellulomonas* sp., *Burkholderia* sp., and *Lelliottia* sp. were reported by 3% of studies each (Table 1).

**Table 1:** Potential calcite dissolving bacteria (CDB) species reported in different studies.

**CDB**	**References**
*Bacillus luciferensis, Bacillus circulans, Bacillus megaterium*	Subrahmanyam et al., 2012; Peper et al., 2022
*Bacillus subtilis*	Friis et al., 2003; Rana et al., 2015; Tamilselvi et al., 2016; Schwantes-Cezario et al., 2017; Baidya et al., 2023
*Bacillus amyloliquefaciens*	Young Nam, 2003
*Brevibacterium* sp. *SOTI06*	Tamilselvi et al., 2018
*Paenibacillus timonensis, Paenibacillus xylanexedens, Paenibacillus etheri, Paenibacillus phocaensis*	Peper et al., 2022
*Buttiauxella warmboldiae, Buttiauxella noackiae, Buttiauxella warmboldiae*	Peper et al., 2022
*Cellulomonas hominis*	Peper et al., 2022
*Enterobacter soli*	Rana et al., 2015; Peper et al., 2022
*Shewanella oneidensis*	Davis et al., 2007
*Burholderia fungorum*	Jacobson and Wu, 2009
*Lelliottia aquatilis, Lelliottia amnegena*	Peper et al., 2022
*Staphylococcus pasteuri, Staphylococcus aureus*	Subrahmanyam et al., 2012; Anbu et al., 2016; Peper et al., 2022
*Pseudomonas aeruginosa*	Anbu et al., 2016; Rana et al., 2015
*Pseudomonas putida*	Baidya et al., 2023
*Sporosarcina pasteurii*	Anbu et al., 2016
*Azotobacter salinestris YRNF3*	Rashad et al., 2023

*Bacillus* sp. was the most reported (29.03%) compared to the other strains, whose efficiency might influence it as a biofertilizer as well as a biocontrol agent, hence widely used compared to others (Agake et al., 2021; Huang et al., 2020). *Bacillus* sp. has been widely used as a biofertilizer and biocontrol against different diseases for different crops and has shown promising results (Huang et al., 2020).

### 3.4 Screening and isolation of CDB

Specific growth media are used in the laboratories to isolate and characterize CDB. According to Rana et al. (2015), preliminary screening and isolation of potential CDB can be done by plating 1 ml of serially diluted rhizospheric suspension on a sterilized calcite agar medium (CDB differentiating medium) supplemented with CaCO_3_ as the only Ca source. As explained by Rana et al. (2015), calcite agar medium composition in grams per liter constitute the following; dextrose 10.0 g, yeast extract 5.0 g, CaCO_3_ 5.0 g, (NH_4_)_2_SO_4_ 0.5 g, KCl 0.2 g, MgSO_4_.7H_2_O 1.0 g, agar 15 g and is adjusted to pH 7.0. Colonies forming a clear halo zone after incubation at an appropriate temperature are screened as CDB. The pure culture of screened CDB colonies is further processed for identification following biochemical and molecular characterization. Ca solubilization ability (Solubilization index-SI) of a specific CDB can be determined by measuring the ratio of the clear zone and colony size on CDB differentiating medium agar plate by using the Equation 2 below (Tamilselvi et al., 2016);


(2) $$\begin{array}{l} \text{Solubilization Index }(\text{SI})\\ =\frac{Diameter\;of\;clear\;zone+diameterof\;colony\;size}{Diameter\;of\;colony\;size} \end{array}$$

### 3.5 Mechanisms of calcite dissolution

Calcite-dissolving bacteria involve various mechanisms to dissolve calcite and make Ca available for plant uptake. These include lowering the pH via organic acid production (acidification), enzyme activity, chelation process, and biofilm formation.

#### 3.5.1 Lowering of the pH via organic acid production (acidification)

The primary mechanism for dissolution and solubilization of Ca is through microbial production of organic acids, which lowers the soil pH (Tamilselvi et al., 2018). Optimal soil-Ca availability in the range of soil pH 6–7.5. Soils with pH below 5.5 and above 7.5 can favor Ca depletion and transformations into plant-unavailable forms, hence hindering its uptake by plants (Goulding, 2016). In alkaline soils, Ca precipitates to form insoluble Ca phosphates, thus making both Ca and P unavailable for plant uptake. Under such soil conditions, CDB increases Ca availability by producing organic acids, which lowers the soil pH to an acceptable range for calcite dissolution (Peper et al., 2022). Organic acids produced by CDB also act as Ca sinks, releasing P from Ca phosphate (a precipitate) and increasing its availability for plant uptake. As the soil pH decreases, the divalent forms of Ca^2+^ increase in the soil. The CDB may release several organic acids (Table 2), which are the results of microbial metabolism, especially the fermentation process when glucose is used as the carbon source (Tamilselvi et al., 2016).

**Table 2:** Diversity of organic acids produced by CDB.

**CDB isolates**	**Organic acids produced**	**References**
*Bacillus subtilis* SSRCI02	Acetic, gluconic, oxalic, lactic, fumaric, propionic, and phytic acids	Tamilselvi et al., 2018
*Brevibacterium* sp. SOTI06	Gluconic, acetic, fumaric, and phytic acids	Tamilselvi et al., 2016
*Enterobacter*	Citric, fumaric, ketoglutaric, malic, and oxalic acids	Rana et al., 2015; Zuluaga et al., 2023
*Pseudomonas* sp	Citric, succinic, fumaric, gluconic and 2-ketogluconic acids	Kalayu, 2019
*Azotobacter salinestris YRNF3*	Lactic, formic, and acetic acids	Rashad et al., 2023

Positive correlation has been reported between Ca solubilization index and organic acid produced, where the maximum solubilization occurs under strong acidic conditions (Tamilselvi et al., 2016). The efficiency of solubilization depends on the strength and nature of the organic acid produced, as revealed by Kalayu (2019), as well as the high chelation effect of the carboxylates detailed under Section 3.4.3. Tri and dicarboxylic acids are more effective compared to monobasic and aromatic acids. Organic acids responsible for Ca solubilization include acetic acid, gluconic acid, oxalic acid, lactic acid, fumaric acid, propionic acid, and phytic acid (Tamilselvi et al., 2016, 2018; Peper et al., 2022). Among these acids, acetic acid is the most predominant, followed by gluconic acid (Tamilselvi et al., 2018). Acetic acid is referred to as the primary organic acid produced by the CDB, such as *Bacillus* sp. (Tamilselvi et al., 2018), while gluconic acid is produced by *Brevibacterium* sp. (Tamilselvi et al., 2016). It has been revealed that Gram-negative bacteria are more effective at solubilizing Ca than Gram-positive bacteria due to the release of diverse organic acids into the environment (Kalayu, 2019; Subrahmanyam et al., 2012). The acidic reaction for the dissolution of calcite is presented in Equation 3 below:


(3) $${\text{CaCO}}_{\text{3}}(\text{s}){\text{+2H}}^{\text{+}}\text{+}(\text{aq})\to {\text{Ca}}^{\text{2+}}(\text{aq}){\text{+H}}_{\text{2}}\text{O }(\text{l})\text{ + }{\text{CO}}_{\text{2}}(\text{g})$$

The released Ca ion (Ca^2+^) becomes available in the soil solution for plant absorption. Besides, the production of organic acids helps the CDB to compete with other microbes by lowering the pH in the environment, which hinders the growth of other bacteria (Rashad et al., 2023).

#### 3.5.2 Enzyme's activity

Calcite-dissolving bacteria such as *Bacillus* sp., *Staphylococcus aureus*, and *Pseudomonas aeruginosa* produce enzymes that play a crucial role in the biogeochemical cycling of Ca. The *Bacillus* sp. produces enzymes such as carbonic anhydrase, which catalyze the hydrolysis process of carbonic acid (Tamilselvi et al., 2016; Subhas et al., 2017). The enzymes produced accelerate the conversion of carbonic acid, bicarbonate ions, and protons, hence promoting the dissolution of calcite by releasing Ca^2+^ into the soil solution (Subhas et al., 2017). Others, such as *Staphylococcus aureus, Pseudomonas aeruginosa, Bacillus megaterium, Bacillus thuringiensis*, and *Sporosarcina pasteurii*, produce urease enzymes which catalyze the hydrolysis of urea and release ammonia, which in turn reacts with dissolved carbon dioxide to form bicarbonate ions that dissolve calcite (Anbu et al., 2016; Baidya et al., 2023). Different studies have reported the capacity of urease to induce carbonate precipitation in microorganisms (Burbank et al., 2012; Stabnikov et al., 2013). Furthermore, urease enzymes play significant roles in the microbial weathering of calcite minerals in the soil (Anbu et al., 2016).

#### 3.5.3 Chelation process

**Figure 3:**
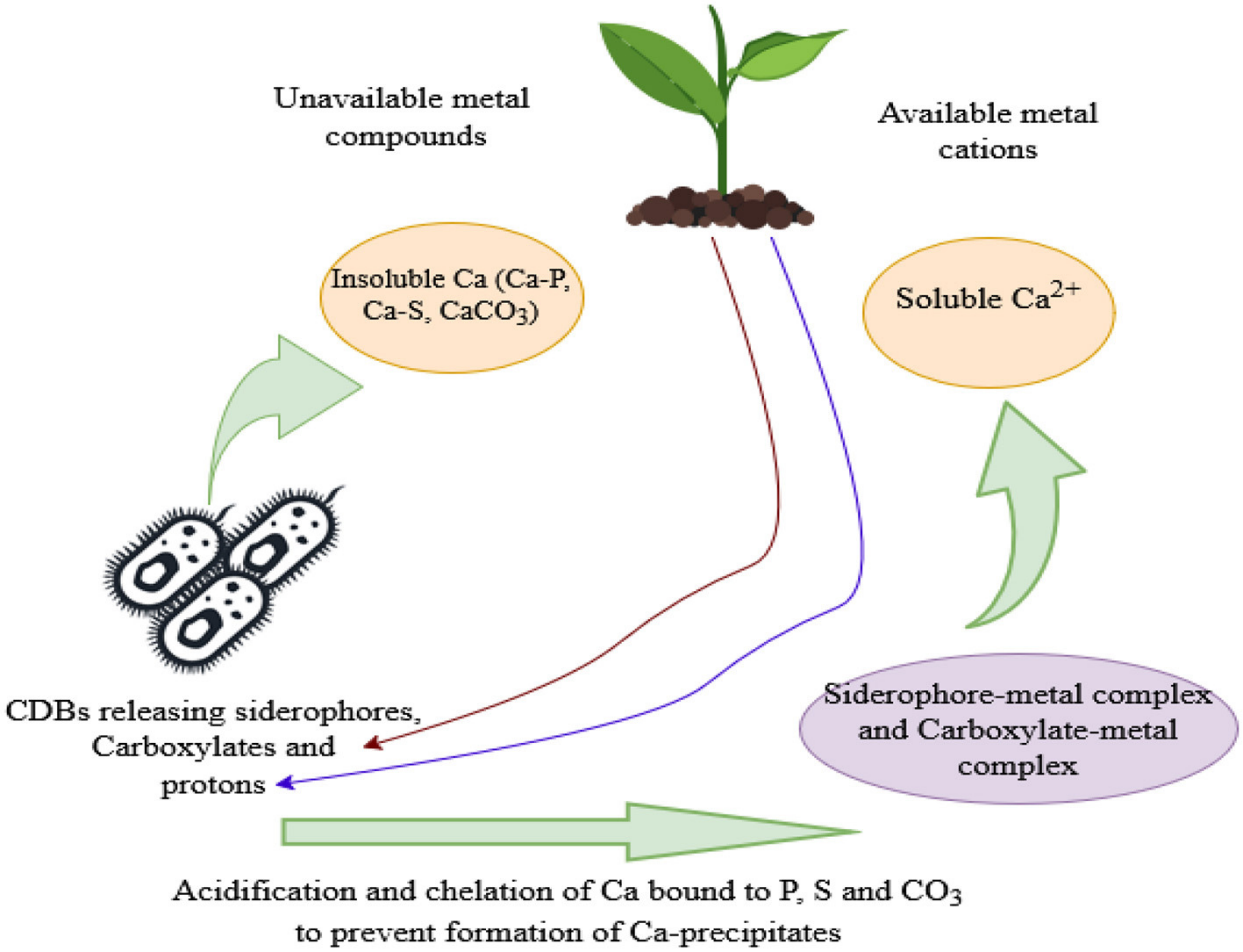
A schematic diagram showing Ca solubilization pathways enhanced by siderophores, carboxylates, and protons released by CDB and plant roots.

Chelation is the formation of multiple coordination bonds between organic molecules and a transition metal ion, leading to sequestration of the metal (Kalayu, 2019). Organic acid anions (carboxylates) and siderophores released by CDB (chelating agents) can act as chelators by dissolving the insoluble soil iron and other metals like Ca and magnesium through their hydroxyl and carboxyl groups, which prevents the cations from precipitating with phosphates, converting them to a soluble form (Pastore et al., 2022; Rashad et al., 2023). Furthermore, the carboxylate-calcium complexes formed enhance the solubility of Ca by lowering the saturation state of Ca in the surrounding environment, hence making it prone to dissolution (Luo et al., 2022). 2-Ketogluconic acid is a powerful chelator of Ca (Kalayu, 2019) as well as lactic acid produced by *Azotobacter salinestris YRNF3* (Rashad et al., 2023). Hence, chelation facilitates the dissolution of calcite and Ca availability for plant uptake (Figure 3).

#### 3.5.4 Biofilm formation

Biofilm is referred to as a community of bacteria colonizing a surface and acting as a multicellular organism (Hall-Stoodley et al., 2004; De Beer and Stoodley, 2006). Microbial biofilms are structurally complex and dynamic systems that can be distinguished from their planktonic counterparts by the presence of an extracellular polymer substance (EPS) matrix, which is the major component of all biofilm organisms (De Beer and Stoodley, 2006). The EPS determines the physical properties of a biofilm, and the constituent bacteria determine the physiological properties of the ecosystem. Calcite-dissolving bacteria such as *Brevibacterium* sp. SOTI06 and *Bacillus subtilis* SSRCI02 can form biofilms on the surface of calcite minerals in the soil, as revealed by Tamilselvi et al. (2016, 2018). These biofilms create a microenvironment that enhances the dissolution of calcite by facilitating the exchange of ions and metabolites between the bacteria and the mineral surface (De Beer and Stoodley, 2006). Biofilm production increases the efficiency of the bacteria in dissolving calcite and releasing Ca^2+^ into the soil solution, making them available for plant uptake.

### 3.6 Plant growth promotion by CDB

Researchers have reported CDB to have the ability to restore the productivity of degraded and unproductive agricultural soil (Backer et al., 2018; Peper et al., 2022). Primarily, CDB enhances plant growth by improving the Ca acquisition efficiency of crops by converting the insoluble forms of Ca into a soluble form, which can easily be accessed and absorbed by different crops, including cereals, horticultural, and perennials (Table 3).

**Table 3:** Effects of CDB on growth yields and performance of different crops.

**CDB**	**Plant**	**Effect induced by CDB on the plant**	**Experiment type**	**References**
*Pseudomonas fluorescent*	Peanut (*Arachis hypogaea* L.)	Increased root length, pod and nodule number, pod set, and pod yield	Pot and field experiment	Dey et al., 2004
*Bacillus*	Peanut (*Arachis hypogaea* L.)	Improved germination rate, pod size, and number of seeds	Pot experiment	Peper et al., 2022
*Pseudomonas*	Maize (*Zea mays*)	Increased plant height, plant biomass, and grain yield	Pot experiment	Bano and Fatima, 2009
*Paenibacillus lentimorbus*	Tobacco (*Nicotiana tabacum)*	Improved flower growth, seeds, and yield	Pot experiment	Kumar et al., 2016
*Bacillus* spp.	Cotton (*Gossypium hirsutum*)	Boost the Gossypol level and jasmonic acid in cotton leaves	Growth chamber	Zebelo et al., 2016
*Pseudomonas putida*	Chickpea (*Cicer arietinum*)	Increased plant height, root length, number of pods, and seed yield.	Pot experiment	Backer et al., 2018
*Bacillus pumilus*	Rice (*Oryza sativa*)	Hastens germination, increases shoot length, fresh weight of shoots, roots, and the whole plant.	Plastic plant box	Agake et al., 2021
*Bacillus pumilus*	Radish (*Raphanus sativus*)	Improved root volume	Field experiment	Aung et al., 2015
*Bacillus subtilis*	Cotton (*Gossypium herbaceum)*	Improved root length and fiber yields	Field experiment	Yao et al., 2006
*Enterobacter hormaechei*	Tomato (*Lycopersicum esculentum*)	Enhanced biomass and shoot length	Hydroponic experiment	Ranawat et al., 2021
*Enterobacter* sp.	Cucumber (*Cucumis sativus*)	Increased shoot and root biomass, as well as leaf area	*In-vitro* experiment	Zuluaga et al., 2023
*Pseudomonas putida*	Tomato (*Lycopersicum esculentum*)	Enhanced plant height, stem diameter, dry biomass, and fruit yield	Pot experiment	Hernández-Montiel et al., 2017
*Pseudomonas putida*	Maize (*Zea mays*)	Increased fresh and dry weight of maize	Pot experiment	Khashei et al., 2020
*Brevibacterium sediminis*	Rice (*Oryza sativa*)	Increased root length, shoot length, and fresh weight	*In-vitro* experiment, pot experiment	Chopra et al., 2020; Mahmud-Ur-Rahman et al., 2022
*Brevibacterium frigoritolerans*	Maize (*Zea mays*)	Advanced root, shoot length, fresh and dry weight of maize	*In vitro* and pot experiment	Batool et al., 2019
*Paenibacillus mucilaginosus*	Green gram (*Vigna radiata*)	Increased sapling length and dry biomass	Pot experiment	Goswami et al., 2015
*Paenibacillus* sp	Wheat (*Triticum aestivum* L)	Enhanced plant height, shoot biomass, and grain yield	Pot and field experiment	Hussain et al., 2020
*Enterobacter aerogenes, Bacillus megaterium*	Wheat (*Triticum aestivum* L)	Improved root, shoot length, and dry weight Increased seed weight	Pot and field experiment	Mukhtar et al., 2017
*Enterobacter* sp	Soybean (Glycine max)	Raised the number of pods, weight of pods, and grain yield	Field experiment	Alkurtany et al., 2023
*Bacillus* sp	Tomato (*Lycopersicum esculentum*)	Increased shoot, root length, fresh and dry biomass Improved antioxidant activity and fruit yield	Pot experiment	Rayavarapu et al., 2016
*Pseudomonas fluorescens*	Tomato (*Lycopersicum esculentum*) and Radish (*Raphanus sativus*)	Increased number of leaves in both tomato varieties and tomato height	*In vitro* and field experiments	Pushpa et al., 2014

Inoculation with CDB, such as the *Bacillus, Brevibacterium*, and *Paenibacillus, Enterobacter* sp., has been reported to increase Ca solubilization in the soil, enhancing crop growth and yields (Backer et al., 2018; Bano and Fatima, 2009; Peper et al., 2022; Subrahmanyam et al., 2012). CDB promotes plant growth via the increase of nutrient availability in the soil for plant uptake, production of phytohormones such as IAA and other auxins, gibberellins, and cytokinins (Backer et al., 2018; Kalayu, 2019). Organic acids produced among the CDB, such as acetic acid, gluconic acid, oxalic acid, lactic acid, fumaric acid, propionic acid, and phytic acid, have been reported to hasten crop maturity by enhancing the straw ratio and total yield of the crops (Charana Walpola, 2012). When applied solely or in combination with other micro-organisms such as Phosphate solubilizers and Nitrogen fixers, CDB has been reported to show considerable effects on plant growth in degraded soil (Bano and Fatima, 2009; Luo et al., 2022). Furthermore, many CDBs are evidenced as effective biofertilizers as well as biocontrol agents, especially the Bacillus, Pseudomonas, and *Paenibacillus* sp (Dey et al., 2004; Huang et al., 2020; Kumar et al., 2016; Zebelo et al., 2016). Hence, calcite dissolution, plant growth promotion, and phytopathogen inhibition effects of CDB make it a potential bioinoculant as a biofertilizer. However, it is worth noting that all studies summarized in Table 3 reported positive findings on the effect induced by CBDs on plant growth and productivity. This may be attributed to publication bias as none of the reviewed studies reported negative effects of CBDs on the same.

### 3.7 Trends and prospects of using CDB as biofertilizer

The efficiency of Ca in farming can be improved via inoculation of CDB. Different studies have revealed the calcite solubilization potentials of CDB (Davis et al., 2007; Jacobson and Wu, 2009; Peper et al., 2022; Tamilselvi et al., 2016, 2018). It has been reported that the inoculation of *Shewanella oneidensis* MR 1, *Burholderia fungorum, Brevibacterium* sp., *Bacillus subtilis, Enterobacter soli*, and *Pseudomonas* sp. show calcite dissolution and increases soil Ca levels compared to uninoculated soil (Davis et al., 2007; Jacobson and Wu, 2009; Peper et al., 2022; Tamilselvi et al., 2016, 2018). Furthermore, it was reported (Peper et al., 2022) that improved Ca uptake, germination, growth, and pod size of peanuts by inoculating *Enterobater soli and Bacillus* species. CDB improves the availability of Ca, but it has minor effects on altering the biochemical composition of the soil, which is principally important for areas with limited access to chemical fertilizers.

Generally, CDB can be used in different crops and is not host-specific. Studies have revealed the potential of CDB in improving growth, yield, and quality of various crops such as peanuts, maize, tomatoes, rice, radish, oranges, and tobacco (Table 3). According to Peper et al. (2022), optimal supply of Ca promotes Ca nutrition, fruit and pod setting, growth, yield, and quality of different crops. It also triggers early ripening and stimulates young plants to produce deeper and abundant roots. Granular calcite is also reported to stimulate natural mycorrhiza associations and the growth of white spruce (*Picea glauca*) seedlings (Lamhamedi et al., 2020). Furthermore, Ca plays a crucial role in the development of cell walls, thereby offering structural support to the plant and impacting its resistance to disease (Huber et al., 2011; Jiang et al., 2013; Shibzukhov et al., 2021). A Bacillus-based inoculant of CDB has been reported to improve barley yields by 43% compared to the control (Chaichi et al., 2008).

Inoculation of peanut seeds with Ca-dissolving *Brevibacterium* isolates increased pod size and weight over the control (Peper et al., 2022). The correlation between the CDB inoculation in the soil with plant height, pod set, number of seeds, biomass production, and Ca was reported (Dey et al., 2004; Peper et al., 2022). Inoculation of *Pseudomonas* isolates, viz. PGPR1, PGPR2, and PGPR4 enhanced peanut pod yield by 23%−26%, 24%−28%, and 18%−24%, respectively, haulm yield, and nodule dry weight over the control for 3 years (Dey et al., 2004). Furthermore, root length, pod number, nodule number, and 100-kernel weight were also improved (Dey et al., 2004). Bano and Fatima (2009) reported that Pseudomonas enhanced maize (*Zea mays*) growth by improving plant height, stem diameter, shoot dry weight, and root length, which increased plant biomass and yield.

A study (Qiu et al., 2021) showed a positive correlation between *Bacillus subtilis* inoculation with fruit internal quality parameters, including the fruit maturity index (FMI), the edible rate, juice yield, and per fruit weight (PFW), and improved fibrous root density in orange. Moreover, the mixed formulation of humic acids with *Pseudomonas fluorescens* indicated dual potentials for crop protection and enhanced growth (Pushpa et al., 2014). Humic acid with *Pseudomonas fluorescens* formulation increases the number of leaves in radish and tomato, as well as tomato height, while it controls wilting caused by *Fusarium oxysporum* in tomato (Pushpa et al., 2014). Furthermore, *Bacillus* sp. *and Glomus monosporum* have been reported to increase tomato growth and antioxidant activity compared to the control. This formulation increases tomato chlorophyll content, proline, and antioxidant enzymes, including catalase, superoxide dismutase, and ascorbate peroxidase (Rayavarapu et al., 2016).

Tomato (*Lycopersicum esculentum*) seeds treated with *Enterobacter hormaechei* showed enhanced biomass, shoot length, and root architecture, leading to improved crop productivity over the control (Ranawat et al., 2021). *Enterobacter colacae* treatment was effective compared to the other treatments in all parameters (Alkurtany et al., 2023). Green gram (*Vigna radiata*) seeds treated with *Paenibacillus mucilaginosus* biofertilizer increase overall dry biomass by 17% and sapling length by 28% compared to the control (Goswami et al., 2015). CDB also promotes plant growth indirectly by increasing the accessibility of trace elements such as Iron and Zinc through siderophore production (Achal and Pan, 2014; Tamilselvi et al., 2016, 2018).

Furthermore, CDB can alternatively act as a biocontrol agent by reducing the occurrence of diseases in plants. It was revealed that the addition of *Bacillus subtilis* suspension reduced the number of second-stage juveniles of RKN on the soil of tobacco seedlings (Huang et al., 2020) as well as inhibition of *Rhizoctonia* sp. and *Macrophomina* sp., hence promoting plant growth and yield enhancement (Tamilselvi et al., 2016). *Brevibacterium frigoritolerans* suppressed Fusarium stalk rot in maize (Batool et al., 2019). *Pseudomonas* sp. suppressed soil-borne fungal diseases such as collar rot of peanut caused by *Aspergillus niger* and stem rot caused by *Sclerotium rolfsii* (Dey et al., 2004). Inoculation with *Peanibacillus lentimorbus* B-30488 in the soil reduced cucumber mosaic virus accumulation in *Nicotiana tabacum* cv. White burley leaves by 91%, which was linked with a rise in stress and pathogenesis-related gene expression and antioxidant enzyme activity, signifying induced resistance against the virus (Kumar et al., 2016). Inoculation of *Bacillus* sp. in Cotton (*Gossypium hirsutum*) increased gossypol and jasmonic acid secretion, which reduces larval feeding by beet armyworm (*Spodoptera exigua)* as revealed by Zebelo et al. (2016).

## 4 Conclusion and recommendations

This study reviewed the potential of using CDB as a biofertilizer to enhance Ca availability and improve crop productivity. The use of CDB inoculants in soil is an efficient way to convert the insoluble mineral Ca to plant-available Ca form, hence positively influencing plant growth, crop yields, and quality of the produce, especially under controlled conditions. The *Bacillus, Brevibacterium, Paenibacillus*, and *Pseudomonas* sp. were the most common and efficient Ca dissolvers capable of enhancing the bioavailability of Ca in the soil. A common mechanism by which CDB promotes immediate plant growth is by increasing plant-available Ca, which enhances plant growth and the plant's resistance to disease pathogens. In this regard, CDB can therefore act as biocontrol agents against different plant pathogens through the production of antibiotics and other metabolites, especially those playing a role in maintaining plant cell walls and integrity of membranes, as well as cell division and elongation. Inoculation of crop seeds with CDB at planting can improve the bioavailability of Ca contained in less soluble mineral compounds within the soil, thus cutting down the need for external Ca inputs, which are often not readily accessible to smallholders. Where available, a combination of mineral Ca with CDB inoculum represents a better option in solving the challenges of Ca deficiency in soils. Furthermore, in areas where smallholder farmers have limited access to commercial fertilizers, soil-Ca availability and removal through crop harvests shall be counterbalanced by application of other Ca inputs, including gypsum, wood ashes, crushed egg shells, and sea shells, compost enriched with Ca-rich materials such as wood ashes, bones, and eggshells. Moreover, crop rotations involving deep-rooted crop plants, as well as adding organic mulches and animal manure, can help mobilize and retain Ca in the soil. These options are environmentally friendly and economically viable, making them potential alternatives in addressing the demand for Ca in plants. Notably, most studies investigating the potential of CDB on enhancing the availability of Ca for plant uptake and crop performance involved pot and hydroponic experiments. Further research is therefore recommended to explore the effectiveness of CDB biofertilizers under field conditions. For a clear understanding of the potential and limitations of using CBD biofertilizers, we recommend avoiding research/publication bias by reporting both positive and negative effects of CBD biofertilizers on plant growth and crop performance.

## Data Availability

The original contributions presented in the study are included in the article/supplementary material, further inquiries can be directed to the corresponding author.
